# Postpartum Vertebral Artery Dissection After Neck Massage Resulting in a Right Cerebellar Infarct: A Case Report and Literature Review

**DOI:** 10.7759/cureus.112847

**Published:** 2026-07-17

**Authors:** Samia Bennani, Zinebe Atlassi, Salma Akkari, Chaimae Amrani, Amal Rami

**Affiliations:** 1 Radiology, Hôpital Universitaire International Cheikh Khalifa, Casablanca, MAR; 2 Radiology, Mohammed VI University of Health Sciences, Hôpital Universitaire International Cheikh Khalifa, Casablanca, MAR

**Keywords:** cerebellar infarct, developmental venous anomaly, hydrocephalus, mri, neck massage, postpartum, radiology, stroke, vertebral artery dissection

## Abstract

Vertebral artery dissection (VAD) can cause posterior-circulation ischemia after cervical manipulation; postpartum physiological changes may increase vascular vulnerability.

A 30-year-old woman, nine months postpartum, developed an abrupt occipital headache, vertigo, nausea, and gait imbalance during a commercial neck massage. On examination, she had truncal ataxia and gait instability without focal limb weakness.

We obtained 1.5-T MRI (diffusion-weighted imaging (DWI)/apparent diffusion coefficient (ADC), T1-weighted imaging (T1), T2-weighted imaging (T2), fluid-attenuated inversion recovery (FLAIR), T2*/susceptibility-weighted imaging (SWI), and postcontrast 3D T1), time-of-flight magnetic resonance angiography (TOF-MRA), and computed tomography angiography (CTA). Imaging showed a right VAD with an intramural hematoma and luminal irregularity, right posterior inferior cerebellar artery (PICA) occlusion, and right-predominant cerebellar infarcts. A posterior fossa developmental venous anomaly (DVA) drained adjacent to the outlets of the fourth ventricle. Cerebellar swelling produced triventricular hydrocephalus with transependymal flow and early tonsillar descent.

After multidisciplinary discussion, we initiated antithrombotic therapy, provided supportive care and a short course of corticosteroids for vasogenic edema, and preserved the DVA drainage. Neurosurgery placed an external ventricular drain for obstructive hydrocephalus; serial imaging documented regression of the edema and normalization of the ventricles. The patient improved but retained mild truncal ataxia at follow-up.

Prompt vascular imaging should be obtained for acute posterior head or neck pain with neurologic signs after cervical manipulation. CSF diversion and edema control should be prioritized when posterior fossa mass effect arises, and DVA drainage should be preserved to avoid venous infarction.

## Introduction

Vertebral artery dissection (VAD), an intimal tear or intramural hematoma of a cervical vertebral artery, produces posterior-circulation ischemia that most commonly affects the cerebellum or brainstem and may rapidly progress to space-occupying posterior fossa edema. Younger adults account for a substantial share of cervical artery dissections; clinicians should suspect dissection in any young patient with new posterior head or neck pain accompanied by acute neurologic signs [1,2]. Case reports and small series have associated vigorous cervical manipulation or neck massage with subsequent VAD, although large population studies remain inconclusive because early dissection symptoms can mimic benign neck pain, and temporal association does not prove causation [3,4]. The postpartum period alters vascular compliance and coagulation and appears repeatedly in dissection series, suggesting transiently increased vulnerability after delivery [5]. Developmental venous anomalies (DVAs) are usually incidental venous variants, but a large posterior fossa collector adjacent to the aqueduct or fourth-ventricle outlets can mechanically worsen CSF outflow when surrounding edema or venous engorgement occurs, risking obstructive hydrocephalus [6]. In this report, we describe a woman nine months postpartum who developed right VAD after a commercial neck massage, right-predominant cerebellar infarction, and transient triventricular hydrocephalus in the setting of a posterior fossa DVA. We correlate vessel-wall and lumen imaging (high-resolution T1-weighted fat-suppressed imaging (T1 FS), time-of-flight magnetic resonance angiography (TOF-MRA), and computed tomography angiography (CTA)) with the clinical course and discuss imaging and management implications for similar cases [7-11].

## Case presentation

A 30-year-old woman, nine months postpartum, developed a sudden severe occipital headache, vertigo, and nausea during a commercial neck massage; she subsequently developed gait imbalance and persistent headache and presented to the ED the following day. On arrival, she was hemodynamically stable; neurologic examination showed truncal ataxia and gait instability without focal limb weakness. Emergency teams obtained 1.5-T MRI with diffusion-weighted imaging (DWI)/apparent diffusion coefficient (ADC), T1, T2-weighted imaging (T2), fluid-attenuated inversion recovery (FLAIR), T2*/susceptibility-weighted imaging (SWI), postcontrast 3D T1, and TOF-MRA and performed CTA to reduce uncertainty related to motion artifact and confirm the vascular findings.

**Figure 1:**
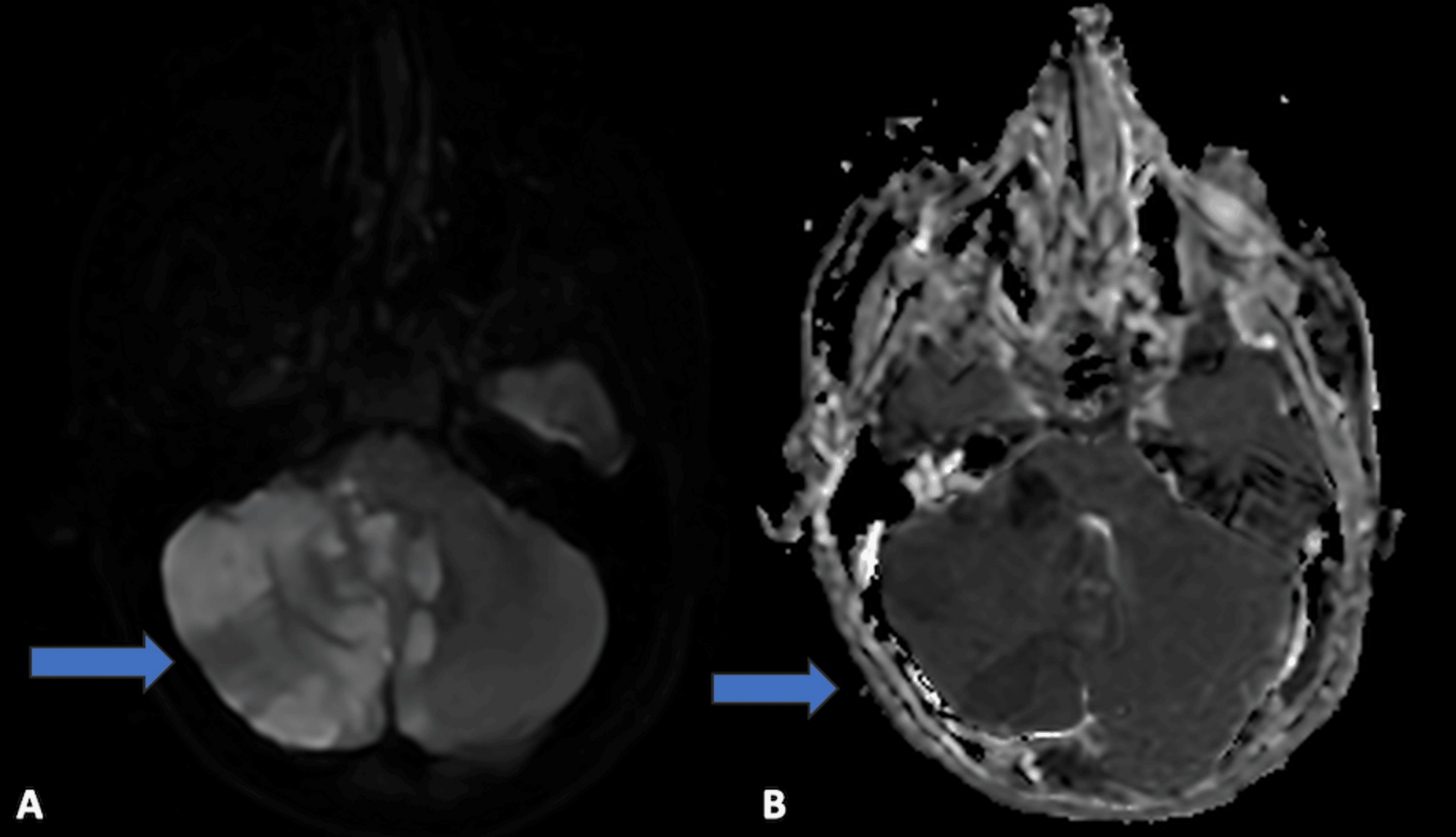
Restricted diffusion on diffusion-weighted imaging (DWI) (A), with corresponding low apparent diffusion coefficient (ADC) signal (B) in the right cerebellar hemisphere, consistent with a focal infarct.

**Figure 2:**
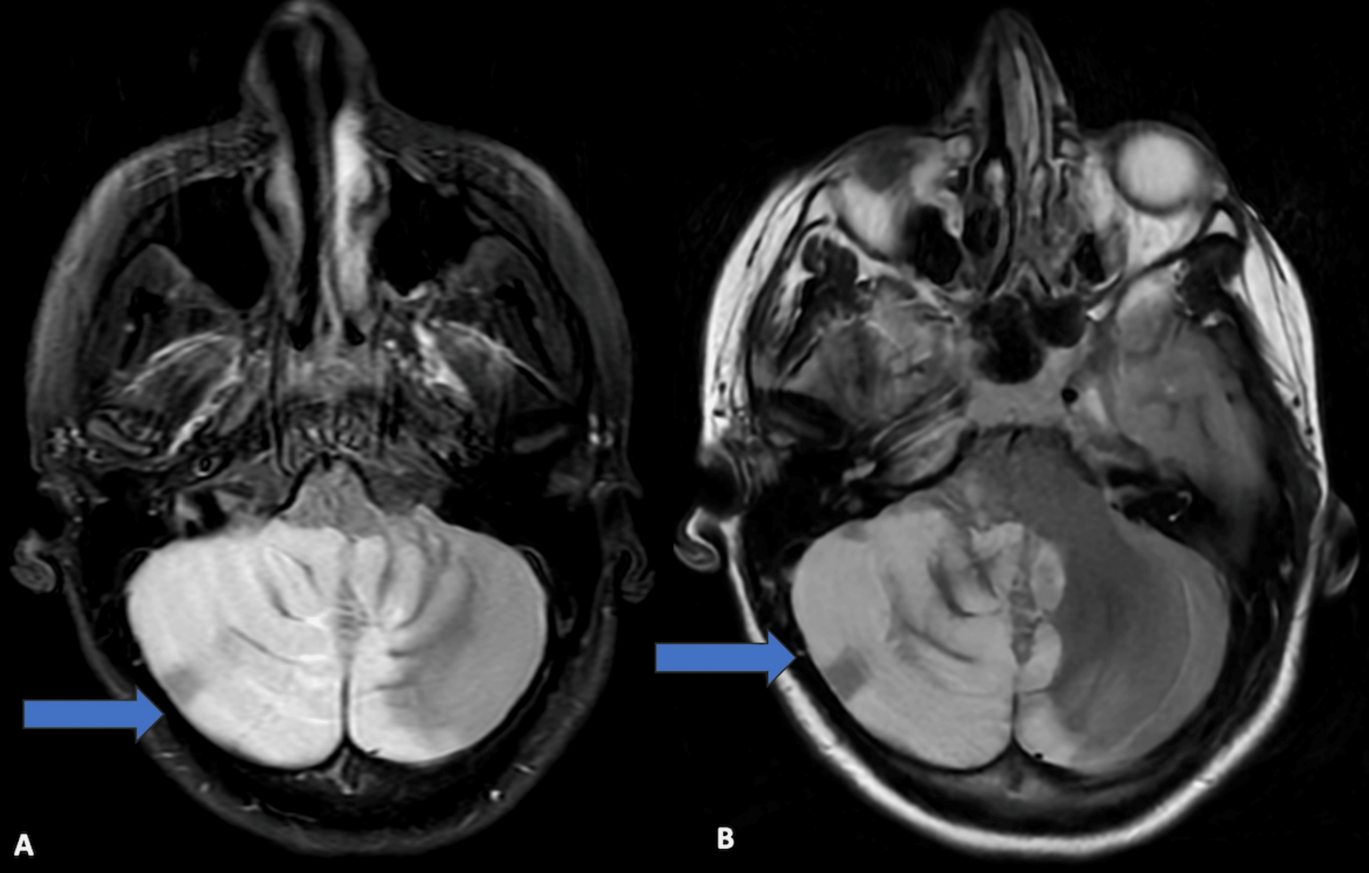
Axial fluid-attenuated inversion recovery (FLAIR) (A) and T2-weighted (B) images showing extensive cerebellar hyperintensity consistent with edema, with effacement of the cerebellar sulci and mass effect on the fourth ventricle.

**Figure 3:**
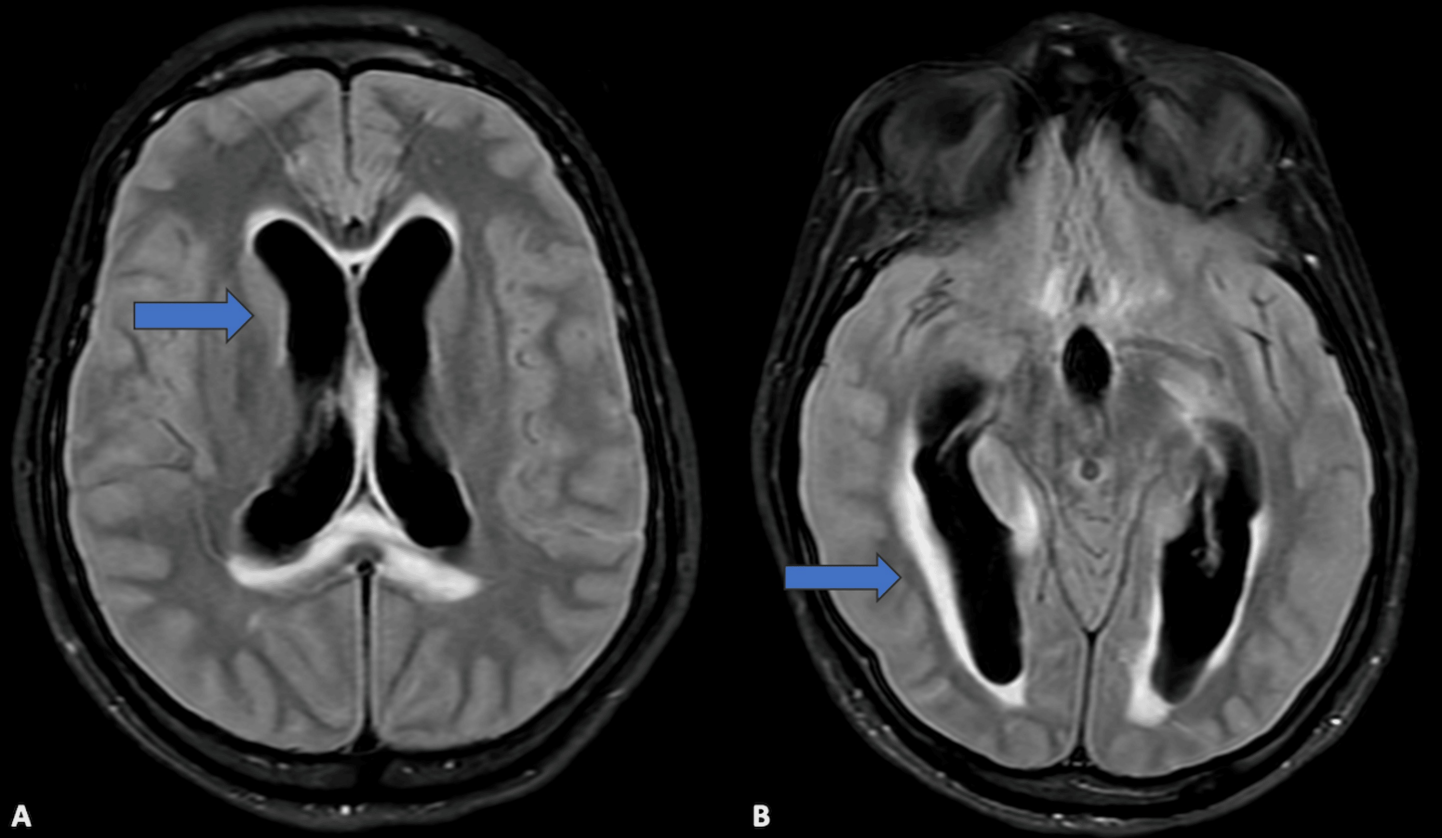
Axial fluid-attenuated inversion recovery (FLAIR) images showing triventricular dilatation (A) with transependymal CSF flow (B).

**Figure 4:**
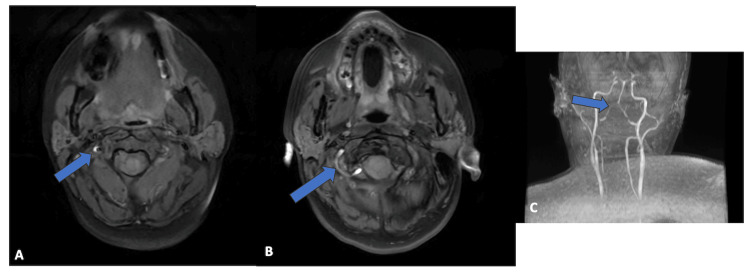
High-resolution T1-weighted fat-suppressed (T1 FS) images (A and B) and 3D time-of-flight magnetic resonance angiography (3D TOF-MRA) image (C) showing eccentric wall hyperintensity and focal thickening of the V3-V4 segments of the right vertebral artery, with luminal narrowing compatible with dissection.

**Figure 5:**
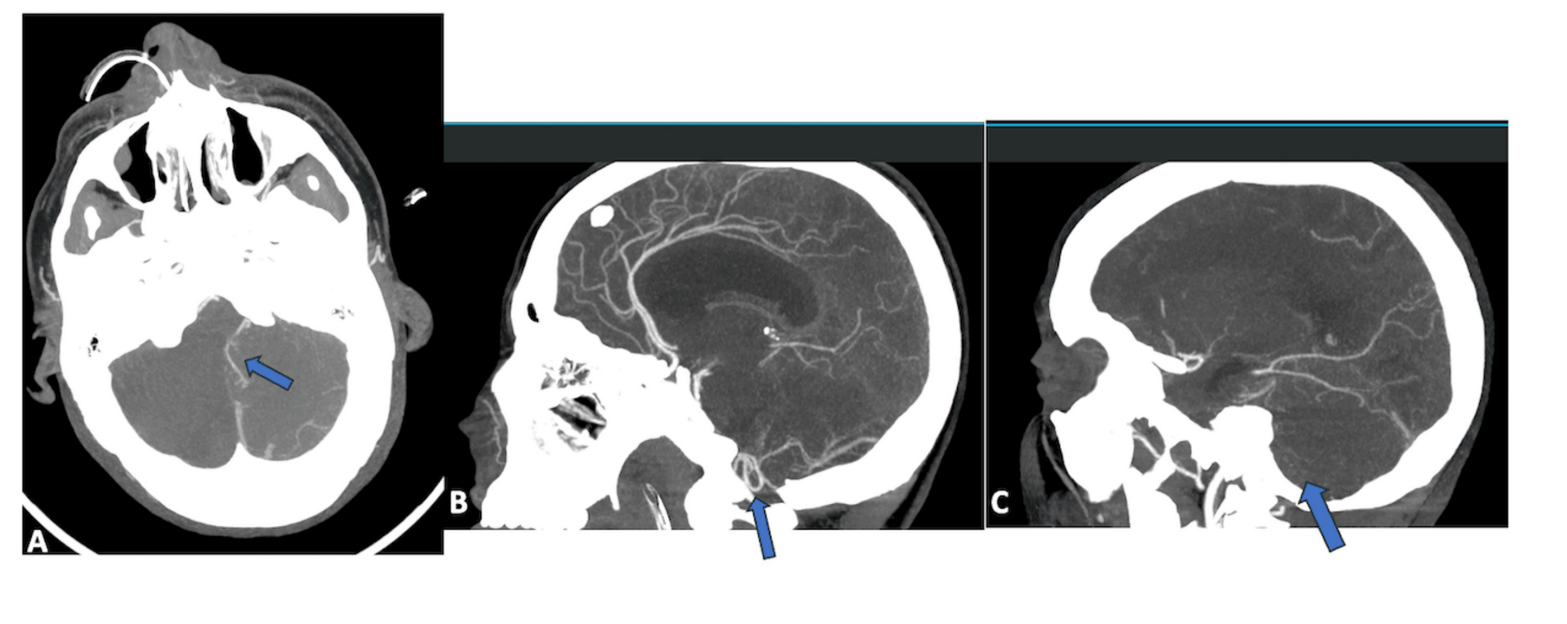
3D time-of-flight magnetic resonance angiography (3D TOF-MRA) axial (A) and sagittal (B) images showing preserved flow in the left posterior inferior cerebellar artery (PICA); the sagittal image (C) shows absent flow with an abrupt cutoff of the right PICA, consistent with occlusion.

**Figure 6:**
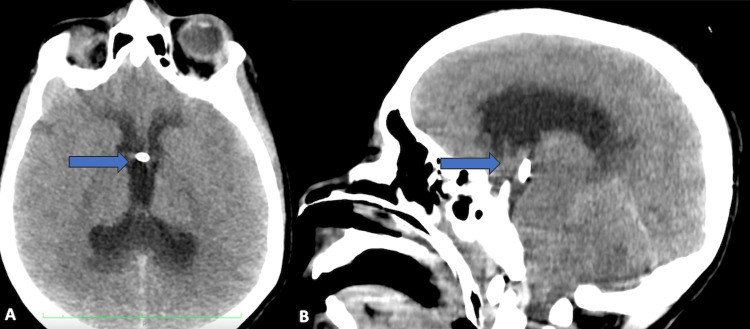
Non-contrast CT images of the head with axial (A) and sagittal (B) reconstructions showing the external ventricular drain (EVD).

Imaging demonstrated acute restricted diffusion in both cerebellar hemispheres, with right-predominant involvement and low apparent diffusion coefficient (ADC), consistent with acute infarction (Figure 1); T2/FLAIR showed cerebellar vasogenic edema compressing the fourth ventricle (Figure 2) and producing active triventricular hydrocephalus with transependymal flow (Figure 3). T2*/SWI showed no hemorrhagic transformation. High-resolution T1 with fat suppression revealed focal eccentric wall thickening and T1-hyperintense intramural signal in the right vertebral artery (V3-V4), consistent with dissection (Figure 4); CTA confirmed right posterior inferior cerebellar artery (PICA) occlusion and right vertebral artery luminal irregularity (Figure 5). We started antithrombotic therapy after multidisciplinary discussion, provided supportive care and a short course of corticosteroids for vasogenic edema, and elected to preserve the posterior fossa developmental venous anomaly that drained adjacent to the fourth-ventricle outlets. Neurosurgery placed an external ventricular drain for obstructive hydrocephalus (Figure 6); subsequent serial imaging showed regression of the cerebellar edema and normalization of the ventricles, and the patient improved clinically, with mild residual truncal ataxia at follow-up.

Clinical course

After multidisciplinary discussion involving neurology, neurosurgery, and neurointerventional radiology, we initiated antithrombotic therapy (300 mg of acetylsalicylic acid) and a short course of corticosteroids to reduce vasogenic edema. The patient required intubation for airway protection and ventilatory support; she was extubated once her respiratory and neurologic status improved. Neurosurgery placed an external ventricular drain for CSF diversion and intracranial pressure (ICP) monitoring; post-external ventricular drain (EVD) CT showed marked ventricular decompression, and follow-up MRI documented progressive regression of cerebellar edema. The team removed the EVD after clinical and radiologic stabilization. At the last assessment, she remained in the ICU on low-flow oxygen via nasal cannula, was conscious and followed commands but was non-verbal, continued daily motor physiotherapy, and underwent ongoing neurologic and imaging surveillance before planned step-down care and outpatient rehabilitation.

## Discussion

VAD typically follows an intimal tear or intramural hematoma that narrows the lumen, promotes thrombus formation, and generates embolic or hemodynamic posterior-circulation ischemia. VAD preferentially involves the V3-V4 segments and commonly produces PICA-territory cerebellar infarcts, as in this case [1,2]. Case reports and small series have linked high-velocity cervical manipulation and vigorous neck massage to subsequent VAD; large population studies remain inconclusive because early dissection symptoms often mimic benign neck pain, and temporal association does not prove causation [3,4]. Our patient’s rapid symptom onset during a commercial neck massage and concordant vessel-wall and lumen findings on high-resolution T1 FS, TOF-MRA, and CTA strengthen the temporal and radiologic association, although causality remains unproven.

Pregnancy and the postpartum period modify vascular compliance, coagulation, and hemodynamics and appear repeatedly in cervical artery dissection cohorts, suggesting transiently increased vascular vulnerability after delivery [1,5]. This case aligns with prior reports that identify the postpartum period as a potential risk state; clinicians should therefore maintain heightened suspicion for cervical artery dissection in young postpartum patients who present with acute posterior head or neck pain and neurologic signs.

Cerebellar infarction produces both cytotoxic and vasogenic edema that can expand rapidly within the tight posterior fossa and obstruct the fourth-ventricle outlets, causing triventricular hydrocephalus, transependymal flow, and a risk of brainstem compression or herniation [6]. Our patient developed clinically significant posterior fossa mass effect requiring CSF diversion. This course is consistent with prior series in which posterior fossa strokes from VAD necessitated urgent neurosurgical intervention; early neurosurgical involvement is critical because timely EVD or decompression can be lifesaving.

DVAs usually remain incidental and require no intervention. However, when a large posterior fossa DVA collector lies adjacent to the aqueduct or fourth-ventricle outlets, adjacent edema or venous engorgement can mechanically worsen CSF outflow obstruction. Case reports document DVA-related obstructive hydrocephalus and warn that DVA sacrifice risks venous infarction; thus, DVA drainage should be preserved, and CSF diversion and edema control should be prioritized over direct venous interventions whenever possible [5,11]. In our patient, we preserved the DVA and used EVD plus edema-targeted therapy, avoiding venous manipulation, an approach consistent with the limited literature and prudent neurosurgical principles.

Imaging strategy influences diagnosis and management. DWI is used to detect acute infarction, FLAIR/T2 to evaluate vasogenic edema and transependymal flow, and SWI/T2* to exclude hemorrhage. High-resolution T1 FS (or vessel-wall/black-blood sequences, when available) should be combined with lumen imaging (TOF-MRA and CTA) to detect intramural hematoma, eccentric wall thickening, focal tapering, or pseudoaneurysm, features that influence antithrombotic decisions and endovascular planning [7-10]. In this case, vessel-wall signal and CTA correlation confirmed a right V3-V4 intramural hematoma with right PICA occlusion; these findings guided the team toward antiplatelet therapy, while the associated hydrocephalus and DVA informed EVD placement and avoidance of DVA intervention.

Therapeutic decisions for cervical artery dissection balance embolic prevention, hemorrhagic risk, and the local mass effect of infarction. Randomized data from CADISS show no clear short-term outcome difference between antiplatelet and anticoagulation therapy for cervical artery dissection; therefore, therapy should be tailored to infarct size, hemorrhagic transformation risk, and multidisciplinary judgment [2]. For posterior fossa swelling with obstructive hydrocephalus, prioritize neurosurgical measures (EVD or decompression) to stabilize ICP and protect the brainstem; manage edema with targeted measures and reserve osmotherapy or decompression based on neurosurgical assessment and hemodynamics. Our team administered a 300-mg aspirin loading dose, used a short course of corticosteroids to address the prominent vasogenic edema on FLAIR/T2, and relied on EVD for dependable CSF diversion, choices made after multidisciplinary risk-benefit assessment.

This single case cannot prove causation between neck massage and VAD and carries the usual limitations of case reports. Vessel-imaging sensitivity depends on sequence and field strength; black-blood and higher-field MRI may improve intramural hematoma detection. Future work should use prospective registries and standardized vessel-wall MRI protocols to clarify postpartum risk, the true association with cervical manipulation, and the role of DVAs in obstructive hydrocephalus [3,4,7-10].

## Conclusions

We report a postpartum patient who developed right VAD after cervical manipulation, resulting in right-predominant bilateral cerebellar infarcts and transient triventricular obstructive hydrocephalus; a nearby posterior fossa DVA likely amplified CSF outflow obstruction. Clinicians should perform vascular imaging in patients with new posterior head or neck pain accompanied by neurologic signs after cervical manipulation, monitor for posterior fossa swelling and hydrocephalus, and manage such cases collaboratively while preserving DVA drainage because its occlusion risks venous infarction.
